# Combined vitamin D and coconut oil are promising protective approaches against aluminum-induced testicular damage in rats

**DOI:** 10.1016/j.toxrep.2025.102051

**Published:** 2025-05-18

**Authors:** Fatma A. Al-Nefeiy

**Affiliations:** Department of Biology, College of Sciences, University of Jeddah, Jeddah, Saudi Arabia

**Keywords:** Seminiferous tubules, Aluminum chloride, Vitamin D. virgin coconut oil, Oxidative stress, Histopathology

## Abstract

Exposure to high levels of aluminum (Al) is a widespread environmental problem where its use is continuously increasing. Al may play a substantial role in male fertility decline. This study was designed experimentally in rats to explore the protective effect of both vitamin D (VD) and virgin coconut oil (VCO) against aluminum chloride (AlCl_3_) on the biochemical and histopathological changes of the testis. Male rats were divided into 7 groups: Control group, VD group, VCO group, AlCl3-treated group, AlCl3 co-treated with VD group, AlCl3 co-treated with VCO group, AlCl_3_ co-treated with both VD and VCO group. After six weeks of treatment, the rats of each group were sacrificed where blood and testicular samples were assembled and processed for different biochemical and histopathological studies. The results showed that AlCl_3_ reduced body weight gain, testis weights, reproductive hormones (FSH, LH, testosterone), and antioxidant enzymes (SOD, GPx, CAT), while elevating oxidative stress (MDA). These effects were notably reversed by VD, VCO, or their combination, with combined therapy showing the greatest improvement. Histologically, AlCl_3_ caused severe testicular damage, including disrupted seminiferous tubules, degenerated spermatogenic cells, interstitial fibrosis, and Leydig cell loss, findings partially restored by VD or VCO and nearly normalized with their combination. Immunohistochemistry further confirmed these improvements, with combined treatment reducing caspase-3 (apoptosis) and restoring Ki-67 (proliferation) expression. In conclusion, AlCl_3_ exposure impaired testicular function by disrupting hormone levels, antioxidant defenses, and tissue integrity. VD and VCO, especially in combination, effectively counteracted these effects, restoring hormonal balance, antioxidant status, and normal tissue structure with reducing apoptosis and enhancing proliferation. Collectively, these findings suggest that VD and VCO synergistically counteract AlCl_3_-induced testicular toxicity, offering anti-inflammatory, antioxidant, anti-apoptotic, and proliferative mechanisms.

## Highlights

• Aluminum exposure harms male fertility.
• Vitamin D and VCO protect testis health.
• AlCl3 induces oxidative stress, inflammation.
• VD/VCO improve hormones, reduce Al levels.
• Combined VD/VCO show stronger protection.

## Graphical Abstract

**Figure:**
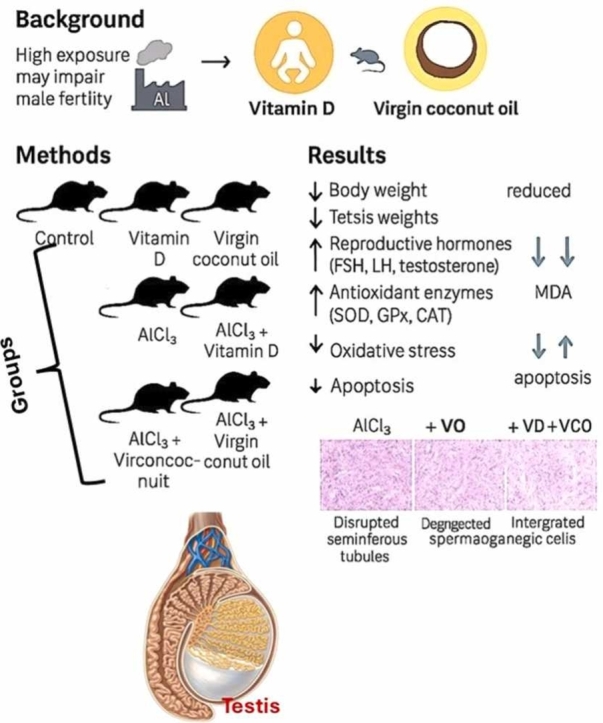

## 1 Introduction

Aluminum (Al) is one of the most abundant metallic elements in the environment, with its release into the atmosphere primarily attributed to industrial and occupational activities. This widespread use has led to a significant increase in environmental aluminum levels, resulting in inevitable human exposure [43]. Additionally, aluminum is extensively utilized across various industries and incorporated into numerous everyday products, including household cookware, storage utensils, cans, foils, food additives, and medications such as antacids, anti-diarrheal drugs, deodorants, and vaccines. This broad application facilitates easy and continuous exposure to Al in human populations [12], [37].

It has been well documented that human exposure to Al occurs primarily through oral intake [45]. Once ingested, aluminum is absorbed through the intestinal mucosal lining and accumulates in various body organs, triggering the development of several diseases, including hematopoietic and respiratory disorders, cardiotoxicity, hepatotoxicity, nephrotoxicity, osteomalacia, and Alzheimer’s disease [6], [15], [25].

The detrimental effects of Al on human reproduction and fertility have been well documented [55]. Al exposure has been linked to toxic impacts on male reproductive organs, including the testes. Several studies have confirmed that exposure to aluminum is associated with testicular tissue damage, impaired spermatogenesis, and disruptions in sex hormone levels [39].

Several mechanisms have been proposed to explain the pathogenesis of Al-induced testicular toxicity, including the promotion of oxidative stress and interference with steroidogenesis. Oxidative stress disrupts the balance between pro-oxidants and antioxidants, leading to the accumulation of reactive oxygen species (ROS) in the testes. This accumulation damages spermatozoa and DNA, impairs Leydig cell function, and results in hormonal imbalances. Additionally, other mechanisms contributing to Al-induced toxicity include the upregulation of inflammatory cytokines, further exacerbating testicular damage [2], [39].

Although several preventive agents, such as chelation therapy, have been employed to reduce Al toxicity [22], they have not proven to be significantly effective and are often associated with adverse side effects [43]. Consequently, there is a growing need to develop safer and more effective natural agents that can protect against and mitigate the continually increasing impact of Al toxicity. In recent years, considerable attention has been directed toward the use of natural antioxidants in managing disorders associated with oxidative stress [7].

In this context, growing evidence suggests that dietary supplements and natural therapies may offer therapeutic benefits for treating a variety of diseases [52]. Both vitamin D (VD) and virgin coconut oil (VCO) have established strong reputations as low-cost, non-pharmacological interventions with protective effects against a wide range of health conditions [9], [49].

VD is typically obtained from dietary sources and is also synthesized in the skin following exposure to ultraviolet (UV) rays [21]. In addition to its well-known role in maintaining calcium balance and skeletal integrity, VD possesses important antioxidant and anti-inflammatory properties [47]. Moreover, VD plays a significant role in supporting spermatogenesis and androgen synthesis within the male reproductive system [13], [41].

Furthermore, VCO is extracted from fresh coconut, which contains many biologically active components of polyphenols, tocopherols and sterols as well as vitamins and minerals. Nowadays, VCO is emerging as functional food oil with numerous applications; having a remarkable role in food, medicine and cosmetics due to its health-promoting properties [18]. The probable advantage of VCO in enhancing several medical conditions is due to its anti-inflammatory, antihyperlipidemic, and antioxidant features [27], [42], [51]. Accordingly, it was decided in this study to investigate both VD and VCO as protective natural agents against the testicular injury induced by aluminum chloride (AlCl_3_) administration to rats.

## 2 Material and methods

### 2.1 Chemicals

**Aluminum chloride (AlCl₃)** was purchased from Sigma-Aldrich (USA), Catalog No. 208407. It was obtained as a white crystalline powder with purity ≥ 99 % (anhydrous).

**Vitamin D** was administered as cholecalciferol (vitamin D₃) in the form of oil-based soft gel capsules, each containing 1000 IU. The product was purchased from Sigma-Aldrich (Catalog No. D1072).

**Virgin Coconut Oil (VCO)** was purchased from Sigma-Aldrich (Catalog No. W440609, Sigma-Aldrich, USA). The VCO was cold-pressed, unrefined, and non-hydrogenated, containing approximately 60–65 % medium-chain fatty acids (MCFAs), with lauric acid as the predominant component (∼50 %), along with capric and caprylic acids.

### 2.2 Animals’ groups and treatment protocol

Mature male Sprague-Dawley rats (200–220 g) were housed separately in plastic cages in adujested environmental conditions (22 ± 3 °C, 50 ± 20 % relative humidity and 12 h light/12 h dark cycles). All rats had a free access to a rodent chow diet and purified water. During all steps of the study, the rats were cared for in the Animal Research Unit, Faculty of Pharmacy, KAU. This study was conducted in compliance with the National Institutes of Health (NIH) *Guide for the Care and Use of Laboratory Animals* (8th edition, 2011) and approved by the Institutional Animal Care and Use Committee (IACUC) of Faculty of Medicine, KAU (reference No. 784–20). After acclimatization to the lab environment for one week, the rats were randomly divided into the following groups (*n* = 8):

– Group I (Control group): the rats received 2.0 ml. of distilled water/day.
– Group II (VD-treated group): the rats received VD at a dose of 200 IU/kg b.wt/day [29]. This dose was selected for its proven ability to mitigate oxidative stress, reduce histopathological damage, and improve reproductive parameters in rodent models of testicular injury.
– Group III (VCO-treated group): the rats received VCO at a dose of 6.7 ml/kg b.wt/day [5]. This dose has been shown to exert antioxidant and anti-inflammatory properties, as well as to ameliorate testicular damage in rodent models without inducing toxicity.
– Group IV (AlCl_3_-treated group): the rats received AlC1_3_ (Sigma, Aldrich (St. Louis, MO, USA) at a dose of 373 mg/kg b.wt/day (This selected dose represents approximately 10 % of the reported oral LD₅₀ (3730 mg/kg) in rats (SIGMA-ALDRICH Safety Data Sheet. Aluminum Chloride. 2013). This sub-lethal dose was chosen to reliably induce biochemical and histological alterations in the testis, without inducing systemic lethality.
– Group V (AlCl_3_ & VD treated group): the rats received VD, one hour before AlCl_3_ administration by the same doses as above.
– Group VI (AlCl_3_ & VCO treated group): the rats received VCO, one hour before AlCl_3_ administration by the same doses as above.
– Group VII (AlCl_3_ & VD & VCO treated group): the rats received both VD and VCO, one hour before AlCl_3_ administration by the same doses as above.

All the treatments were applied orally by using a metallic gastric tube. The rats in each group were closely monitored daily for the body weight in addition of some physical signs of toxicity, which include locomotor activity, tremors, and drowsiness. After six weeks of treatments, the rats of different groups were weighed and anesthetized (using 60 mg/kg ketamine hydrochloride and 6 mg/kg xylazine intramuscularly) where the blood was collected from the retro-orbital venous plexus and centrifuged at 3000 rpm (15 min) to separate the sera, which were stored at −80°C until assayed. Afterwards, the rats were euthanatized by decapitation where the abdomen was incised where the testes were quickly removed and washed, then weighted. Each testis was cut into transverse pieces. Some pieces were immersed quickly kept at −70 °C for later biochemical studies. The rest of pieces were fixed in 10 % neutral buffer formalin (NBF) for 48 h for histological and immunohistochemical assessment.

### 2.3 Assessment of body weight gain and testis weight

The initial and final weights for each rat in different groups were determined to calculate the weight gain using the following formula: Body weight gain = Final body weight – initial body weight. Also, the relative testis weight was calculated as follows: Absolute testis weight/final body weight × 100.

### 2.4 Serum hormonal assay

The serum concentrations of luteinizing hormone (LH), follicle-stimulating hormone (FSH) and testosterone were determined using ELISA kits (Elabscience Biotechnology Co., Ltd., Germany) according to the manufacturer's instructions. Level of testosterone was expressed as ng/ml.

### 2.5 Testicular homogenate and determination of oxidative and antioxidant parameters

The defrosted testis pieces were homogenized in 10 ml ice-cold phosphate buffer saline using Glas-Col, motor-driven homogenizer. Then centrifuged at 3000 rpm at 4 °C for 15 min, and the supernatants were separated for determination of the following oxidative and antioxidant markers using standard kits according to the manufacture’s instructions: The Malondialdehyde (MDA) concentration as a marker of lipid peroxidation (expressed as nmol per gram /tissue). The Superoxide Dismutase (SOD) activity (expressed as U/g tissue). Glutathione Peroxidase (GPx) activity (expressed as U/g tissue). Catalase (CAT) activity (expressed as nmol/g tissue).

### 2.6 Measurement of inflammatory markers in testicular tissue

The levels of nuclear factor kappa B (NF-κB [Catalog No. MBS453975, MyBioSource, USA]), tumor necrosis factor-alpha (TNF-α [Catalog No. RAB0480, Sigma-Aldrich, USA]), interleukin-1 beta (IL-1β [Catalog No. BMS630, Invitrogen, USA]), and interleukin-6 (IL-6 [Catalog No. RAB0312, Sigma-Aldrich, USA]) in testicular tissue homogenate were determined by ELISA analysis (BioAssay Systems, Hayward, CA, USA) following the manufacturer’s protocols.

### 2.7 Estimation of the aluminum concentration

The Al concentration in testis was assessed according to Zhu et al. [58] using PerkinElmer Atomic Absorption Spectrometer (AAnalyst 400, USA). Al concentration was expressed as ng/mg.

### 2.8 Histological and immunohistochemical study

The NBF fixed ovarian pieces were processed using automatic tissue processor at the histology lab (Leica TP1020) to obtain paraffin blocks. Then, 5*μm* thick sections were cut using a rotary microtome, and stained with haematoxylin and eosin (H & E) to assess the general structure and Masson's trichrome (MT) for staining the collagen fibers [10]. Also, immunohistochemistry was achieved as described previously [23] where some slides were processed for immunostaining of ki-67 and caspase-3 antibodies using the streptavidin–peroxidase method with immunoperoxidase kits (Histostain-Plus Bulk Kit; Zymed, South San Francisco, CA, USA) in accord with manufacturer’ instructures. Finally, capturing images of slides from various groups at different magnifications was accomplished using an Olympus BX53 microscope equipped with a camera (Olympus, Tokyo, Japan) at the Department of Clinical Anatomy, Faculty of Medicine, KAU.

### 2.9 Histomorphometric study

The measurements and quantification were done by Image J analyzer software 150e (Bethesda, USA). The measurements were taken from 6 non overlapping fields, and the average was calculated for the following parameters [17]:

1. Diameter (μm) of seminiferous tubules (STs) in H & E-stained slides.
2. Height of the epithelium (μm) of STs in H & E-stained slides.
3. Area % of fibrous tissue in MT- stained slides.
4. Percentage (%) of Ki-67 and caspase-3 immuno-expression in spermatogenic of STs.

## 3 Results

### 3.1 Clinical observations

Throughout the experimental period, the rats were carefully monitored for general health status and clinical signs of systemic toxicity. No overt signs of acute systemic toxicity, such as piloerection or mortality were recorded. However, some signs of abnormal locomotor activity, lethargy and tremors were observed in some rats of AlCl_3_-treated group.

### 3.2 Weight parameters

**Fig. 1:**
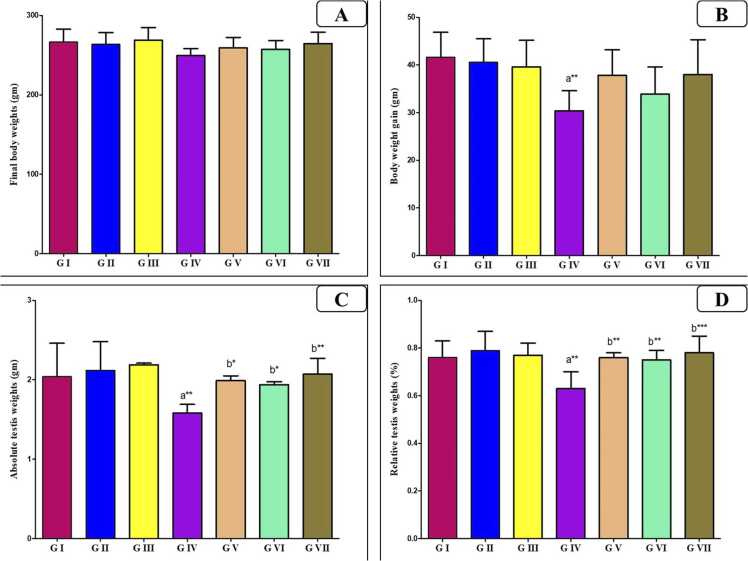
Bar charts showing the statistical comparison of the mean values ± SD of weight parameters between different studied groups (*n* = 8): (A) Final body weight, (B) Body weight gain, (C) Absolute testis weight, (D) Relative testis weight. a: Significance versus GI, b: significance versus GIV. * : P < 0.05, * *: P < 0.01, * ** : P < 0.001. GI = control group, GII = VD treated group, GIII = VCO treated group, GIV = AlCl_3_ group, GV = AlCl_3_ and VD group, GVI = AlCl_3_ and VCO group, GVII = AlCl_3_, VD and VCO group.

Fig. 1 illustrates that there was insignificant variation in the initial body weight among the groups (p > 0.05). Also, there was insignificant variation (p > 0.05) in measured final body weight, body weight gain, absolute testis and relative testis weight between the control, VD and VCO treated groups. In contrast, AlCl_3_ caused a substantial decrease in the body weight gain (P < 0.0001), absolute and relative testis weight (P < 0.010) compared to the control group. Conversely, the concurrent treatments of VD or VCO with AlCl_3_ returned these weights closely towards the control values; being significant compared to AlCl_3_ group (P < 0.05, P < 0.05 and P < 0.010). Nevertheless, the combined administration of both agents to AlCl_3_ group produced a more significant progress (P < 0.010), which was closely similar to the control values.

### 3.3 Serum hormone levels

**Fig. 2:**
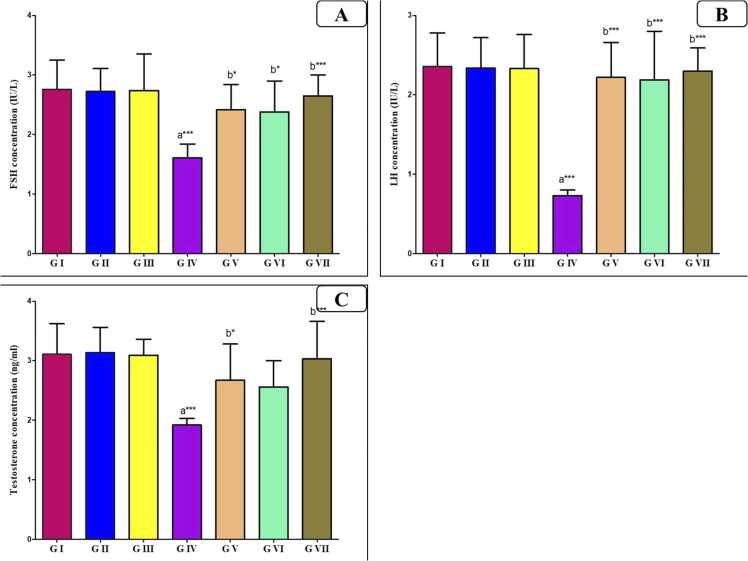
Bar charts showing the statistical comparison of the mean values ± SD of sex hormons parameters between different studied groups (*n* = 8): (A) FSH, (B) LH, (C) Testosterone. a: Significance versus GI, b: significance versus GIV. * : P < 0.05, * *: P < 0.01, * ** : P < 0.001. GI = control group, GII = VD treated group, GIII = VCO treated group, GIV = AlCl_3_ group, GV = AlCl_3_ and VD group, GVI = AlCl_3_ and VCO group, GVII = AlCl_3_, VD and VCO group.

In Fig. 2, the FSH, LH, and testosterone levels showed a significantly low value (P < 0.0001) in the AlCl_3_ treated group compared to the control group. As VD or VCO was administered with AlCl_3_, there was a substantial increase (P < 0.05, P < 0.05) in these hormones toward control values compared to the AlCl_3_ group. Likewise, the AlCl_3_ group's level of these hormones was more effectively returned to normal following the combination treatment of both agents (P < 0.0001).

### 3.4 Oxidative markers and antioxidant enzymes

**Fig. 3:**
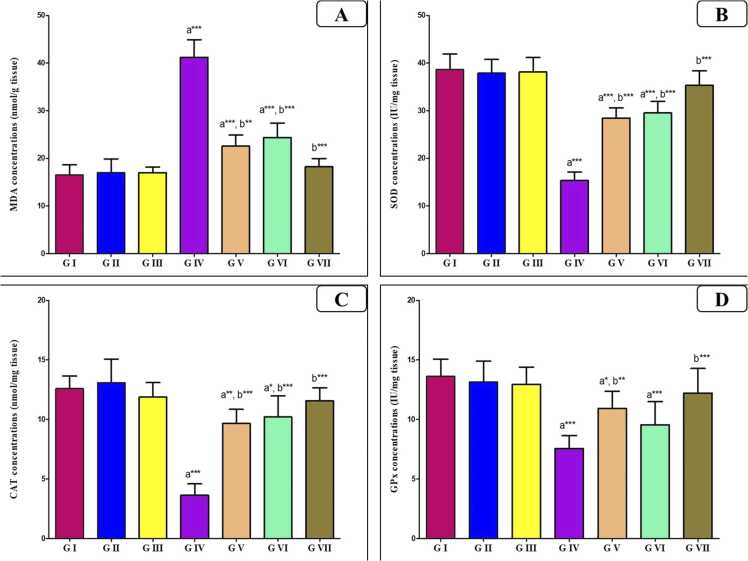
Bar charts showing the statistical comparison of the mean values ± SD of oxidative stress parameters between different studied groups (*n* = 8): (A) MDA, (B) SOD, (C) TCAT, (D) GPx. a: Significance versus GI, b: significance versus GIV. * : P < 0.05, * *: P < 0.01, * ** : P < 0.001. GI = control group, GII = VD treated group, GIII = VCO treated group, GIV = AlCl_3_ group, GV = AlCl_3_ and VD group, GVI = AlCl_3_ and VCO group, GVII = AlCl_3_, VD and VCO group.

As observed in Fig. 3, AlCl_3_ administration elevated lipid peroxidation represented by a marked rise of MDA level (p < 0.0001) associated with significant depletion (p < 0.0001) of the antioxidant enzymes (SOD, GPx and CAT) as compared to the control groups. Meanwhile, the administration of VD or VCO with AlCl_3_ led to an obvious drop (P < 0.001) in MDA accompanied by elevation of the antioxidant enzymes (P < 0.001) near the control values; being significant compared to Al group. Also, the combined intake of both agents was more effective (p < 0.0001) in the normalization of these parameters.

### 3.5 Results of inflammatory markers in testicular tissue

**Fig. 4:**
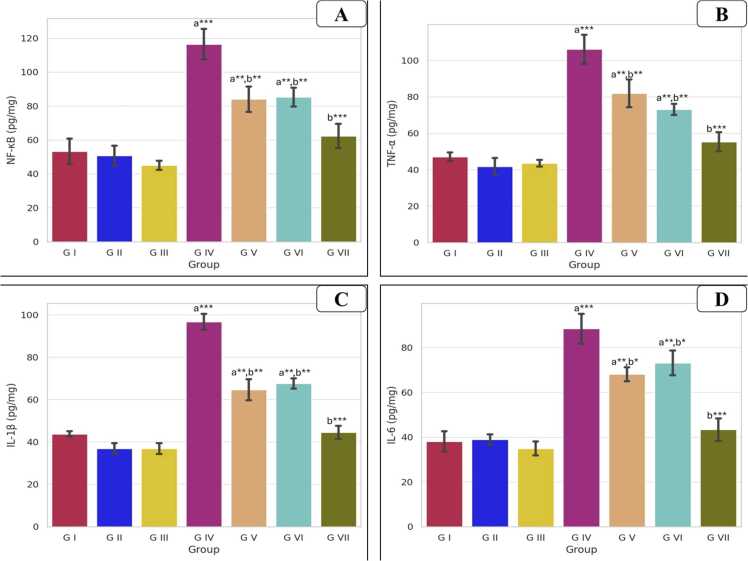
Bar charts showing the statistical comparison of the mean values ± SD of inflammatory markers in testicular tissue between different studied groups: (A) NF-kB (B) TNF-α, (C) IL-1β, (D) IL-6. a: Significance versus GI, b: significance versus GIV. * : P < 0.05, * *: P < 0.01, * ** : P < 0.001. GI = control group, GII = VD treated group, GIII = VCO treated group, GIV = AlCl_3_ group, GV = AlCl_3_ and VD group, GVI = AlCl_3_ and VCO group, GVII = AlCl_3_, VD and VCO group.

As seen in Fig. 4, AlCl_3_ group demonstrated significantly elevated levels of inflammatory markers, including NF-κB, TNF-α, IL-1β, and IL-6, when compared to the control group (p < 0.001) for all markers. However, in the groups of AlCl_3_ co-treated with VD or VCO, there was moderate decline of these markers (p < 0.01) as compared to the AlCl_3_ group. Moreover, the combined intake of both agents to AlCl_3_ group resulted in a significant decrease in these markers as compared to the AlCl_3_ group.

### 3.6 Testicular concentration of Al

**Fig. 5:**
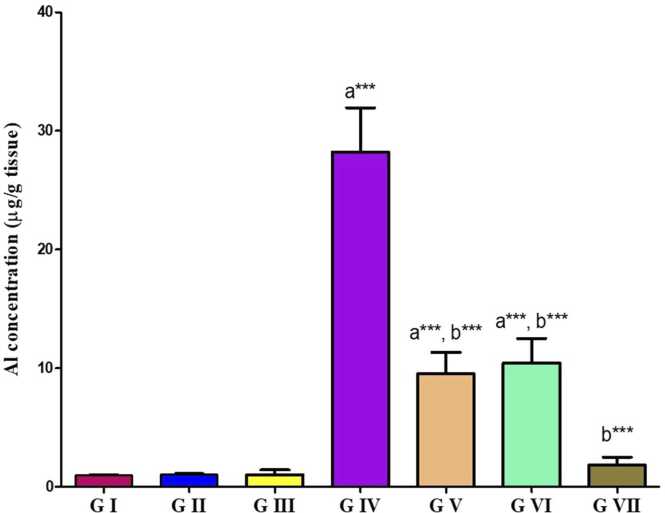
Bar chart showing the statistical comparison of the mean values ± SD of Al concentration between different studied groups (*n* = 8). a: Significance versus GI, b: significance versus GIV. * : P < 0.05, * *: P < 0.01, * ** : P < 0.001. GI = control group, GII = VD treated group, GIII = VCO treated group, GIV = AlCl_3_ group, GV = AlCl_3_ and VD group, GVI = AlCl_3_ and VCO group, GVII = AlCl_3_, VD and VCO group.

As compared to control groups, Al concentration (Fig. 5) in the testicular tissue of the AlCl_3_-treated group was significantly higher (P < 0.0001). In contrast to the AlCl_3_ group, the co-administration of VD or VCO with AlCl3 significantly reduced Al concentration around the control range (p < 0.01). Furthermore, the AlCl3 group had a more substantial improvement after receiving both extracts together, which was roughly equal to the control value (p < 0.001).

### 3.7 Histological results

#### 3.7.1 H&E staining

**Fig. 6:**
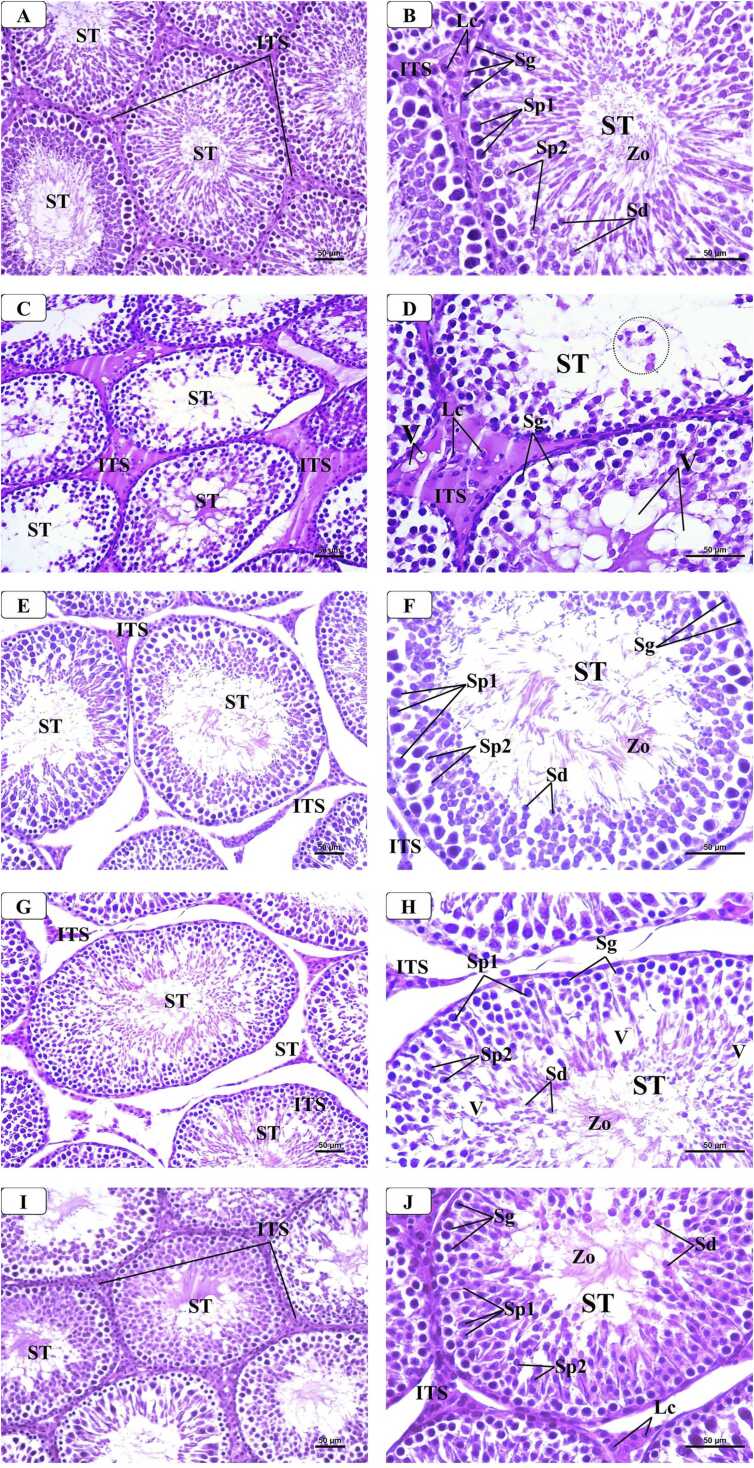
Representative Photomicrographs of H&E-stained sections of the testis from different groups: (A & B) Control group showing normal structure of the testis, formed of regular seminiferous tubules (STs) with spermatozoa (Zo) filled lumina and narrow interstitial spaces (ITS) containing little connective tissue and Leydig cells (Lc). High magnification of STs showed regularly arranged spermatogenic cells formed of spermatogonia (Sg), primary spermatocytes (Sp1), secondary spermatocytes (Sp2), spermatids (Sd). (C & D) AlCl3 treated group showing disorganized, irregular STs, which appeared atrophic and widely separated. The interstitial tissue (ITS) was wide and contained hyalinized interstitial tissue, few degenerated Leydig cells (Lc) and some vacuoles (V). High magnification showed degenerated shrunke spermatogenic cells separated by multiple vacuoles (V) and lumina containing exfoliated cells (circle) with no mature spermatozoa. (E & F) AlCl_3_ with VD co-treated group showing improved testicular structure as compared to AlCl3 treated group. However, some STs showed irregular outlines with wide interstitial spaces (ITS) containing a smaller number of Leydig cells. High magnification showed diminished layers of spermatogenic cells wih wide lumen that contained few spermatozoa (Zo). (G & H) AlCl_3_ with VCO co-treated group showing improved testicular structure as compared to AlCl3 treated group. However, some STs appeared irregular with wide interstitial spaces (ITS) containing a smaller number of Leydig cells. High magnification showed diminished layers of spermatogenic cells separated by multiple vacuoles (V) and few spermatozoa (Zo) in the lumen. (I & J) AlCl_3_ with VD and VCO co-treated group showing that the testicular structure was quite similar to that in control group in the form of regular STs separated by narrow interstitial spaces (ITS) containing normal Leydig cells (Lc). High magnification of STs showed regularly arranged spermatogenic cells in different stages of development and narrow lumen filled with mature sperms (Zo). [H & E stain: A, C, E, G, I × 200 - B, D, F, H, J × 400].

In the control, VD-, and VCO-treated groups (Fig. 6 A & B), un almost identical results of normal structure as regular, closely packed seminiferous tubules (STs) spaced by a little quantity of interstitial tissue. Most of STs displayed a lining of spermatogenic cells in different stages of spermatogenesis that appeared normal and orderly arranged from the basement membrane to the lumen (spermatogonia, large 1^ry^ spermatocytes, 2^ry^ spermatocytes, spermatid, and the mature sperms in the lumen). Blood vessels and Leydig cells, which were big cells with eosinophilic cytoplasm and a single spherical nucleus, were present in the interstitial spaces.

In the AlCl_3_-treated group, the examination showed apparent histological change in the STs and interstitial tissue when compared with control group (Fig. 6 C & D). Many STs appeared distorted and disorganized with loss of the typical architecture. Some tubules were widely separated and looked shrunken with irregular outline. In some tubules, there was a diminishing of layers of spermatogenic cells with widening of the lumina and lack of mature sperms. These cells appeared distorted and showed the presence of irregular vacuoles. Moreover, in the interstitial spaces, hyalinized material was observed and the Leydig cells were degenerated with pyknotic nuclei.

In the AlCl_3_, co-treated with either VD (Fig. 6 E & F) or VCO group (Fig. 6 G & H), showed variable degrees of improvement in both STs and interstitial tissue. Although many STs appeared normal in structure with regular contour and organized lining layers of spermatogenic cells with spermatozoa in their lumen with no detectable degenerative changes, a few to moderate numbers of STs showed degenerated spermatogenic cells and wide lumina containing deformed spermatozoa. Additionally, the interstitial spaces displayed less congested blood vessels and the hyalinized material with normally appearing Leydig cells.

In the AlCl_3_, co-treated with both VD and VCO group, the examination showed marked improvement in both STs and interstitial tissue (Fig. 6 I & J) where STs appeared almost like those of the control testis in the form of regular arrangement and normally lining of spermatogenic and Sertoli cells with their lumina full of spermatozoa. Also, narrow interstitial spaces with an increase of normally appearing Leydig cells and decreased congestion and dilatation of blood vessels were observed.

#### 3.7.2 Masson trichrome (MT) staining

**Fig. 7:**
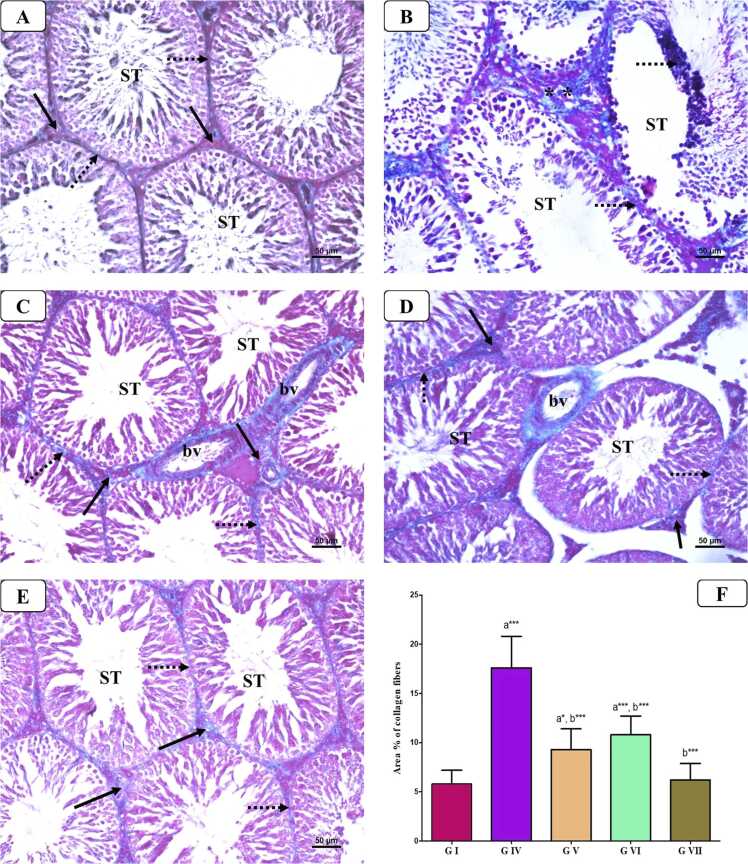
Representative photomicrographs of MT staining of the testis from different groups: A) Control group showing minimal amount of collagen fibers in the interstitial tissues (arrow) and basal lamina of seminiferous tubules (STs) (dotted arrow). B) AlCl_3_-treated group showing a marked increase of the collagen fibers deposition in the interstitial tissues (**) and basal lamina of STs (dotted arrow). C) AlCl_3_ with VD co-treated group showing less deposition of collagen fibers in the interstitial tissues (arrow), around the blood vessels (bv) and in basal lamina of seminiferous tubules (STs) (dotted arrow). D) AlCl_3_ with VCO co-treated group showing weak deposition of collagen fibers in the interstitial tissues (arrow), around the blood vessels (bv) and in basal lamina of seminiferous tubules (STs) (dotted arrow). E) AlCl_3_ with VD and VCO co-treated group showing minimal amount of collagen fibers in the interstitial tissues (arrow) and basal lamina of seminiferous tubules (STs) (dotted arrow). F) Statistical analysis of area % of collagen fibers. [MT x 200].

In MT-stained testis sections (Fig. 7), control groups exhibited minimal fibrous tissue in the interstitial spaces and between STs. In contrast, the AlCl_3_-treated group showed a large amount of fibrous tissue in these areas. However, when AlCl_3_ was co-administered with either VD or VCO, a moderate amount of fibrous tissue was detected. Also, the statistical analysis of the area % of collagen fibers revealed a marked rise in AlCl_3_ treated group (P < 0.0001) compared to that in control group, whereas there was a noticeable reduction in this % in the groups of AlCl3 co-treated with VD and VCO compared to AlCl_3_ treated group (P < 0.01). Furthermore, combined VD and VCO co-treatment to AlCl_3_ group restored the % to back to the control value (P < 0.001).

#### 3.7.3 Immunohistochemical findings

##### 3.7.3.1 Casepase-3 expression (apoptosis marker)

**Fig. 8:**
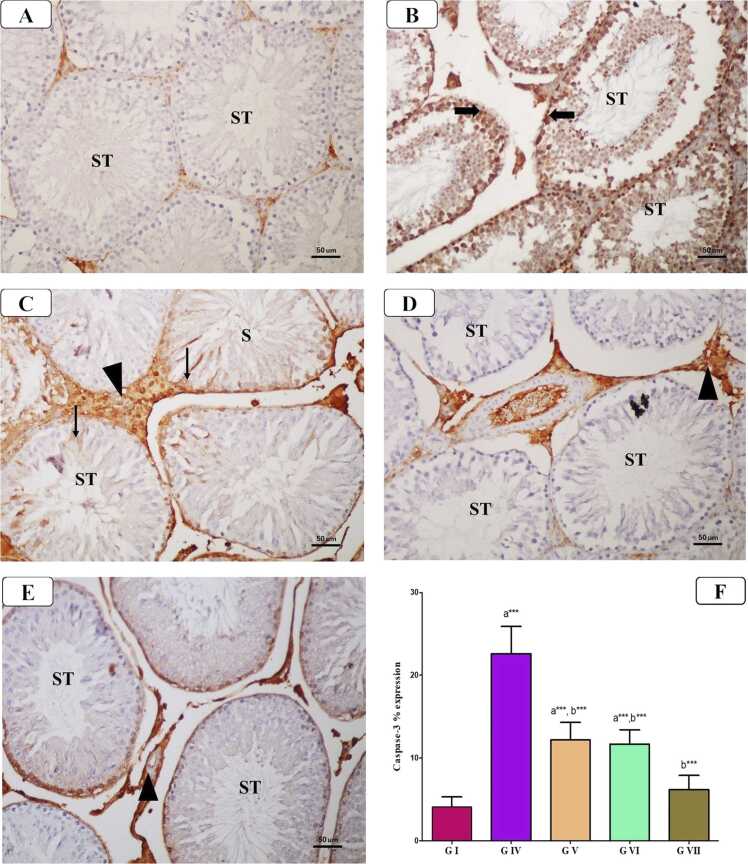
Representative photomicrographs of caspase-3 immunohistochemistry of the testis from different groups: A) Control group showing negative cytoplasmic caspase-3 immunostaining in the spermatogenic cells of seminiferous tubules (STs). B) AlCl_3_-treated group showing diffuse strong positive cytoplasmic caspase-3 immunostaining in the spermatogenic cells of STs (thick arrow). C) AlCl_3_ and VD-treated group showing weak positive cytoplasmic caspase-3 immunostaining in the spermatogenic cells of STs (arrow). Notice the presence of non-specific staining in the interstitial spaces (arrowhead). D) AlCl_3_ and VCO-treated group showing negative caspase-3 immunostaining in the spermatogenic cells of STs with non-specific staining in the interstitial spaces (arrowhead). E) AlCl_3_, VD and VCO-treated group showing negative caspase-3 immunostaining in the spermatogenic cells of STs. F) Statistical analysis of the immuno-expression % of caspase-3 in spermatogenic cells [casepase-3 immunostaning x 200].

In the control group (Fig. 8), there was a negative caspase-3 immunostaining in the cytoplasm of spermatogenic cells. While AlCl_3_ group displayed a substantial rise in caspase-3 immuno-expression in these cells as compared to that in the control groups, indicating cell death. On the other hand, weak caspase-3 expression was seen in the cytoplasm of these cells in AlCl_3_ group co-treated with VD or VCO. However, co-administration of VD and VCO with AlCl_3_ exhibited mild caspase-3 immunostaining closely like the normal control. Also, the statistical analysis of the immuno-expression % of caspase-3 in spermatogenic cells revealed a marked rise in AlCl_3_ treated group (P < 0.0001) compared to that in control group, whereas there was a significant reduction in this % in the groups of AlCl_3_ co-treated with VD and VCO comparing to AlCl_3_ treated group (P < 0.01). Furthermore, combined VD and VCO co-treatment to AlCl_3_ group restored the % to back to the control value (P < 0.001).

##### 3.7.3.2 Ki-67 expression (nuclear-associated antigen Ki67)

**Fig. 9:**
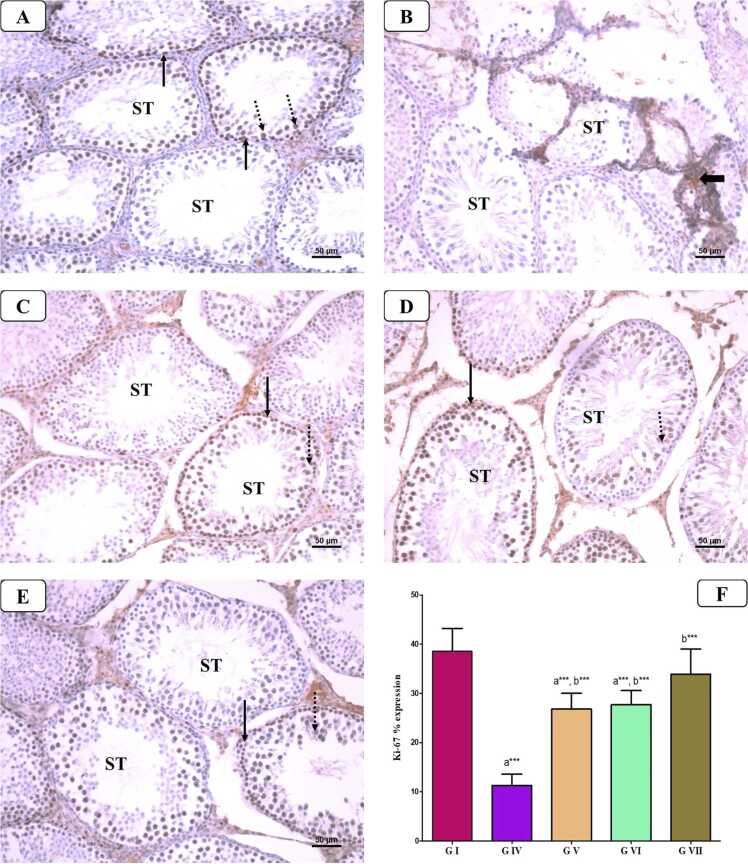
Representative photomicrographs of Ki-67 immunohistochemistry of the testis from different groups: A) Control group showing strong expression of Ki-67 in nuclei (brown color) of spermatogonia (arrow) and spermatocytes (dotted arrow) in the seminiferous tubules (STs). B) AlCl_3_-treated group showing absent expression of Ki-67 in most nuclei of spermatogonia and spermatocytes of STs. Thick arrow referred to non-specific staining in the interstitial spaces. C) AlCl_3_ and VD-treated group showing moderate increased expression of Ki-67 in the nuclei of spermatogonia (arrow) and spermatocytes (dotted arrow) of STs. D) AlCl_3_ and VCO-treated group showing an increased expression of Ki-67 in the nuclei of spermatogonia (arrow) and spermatocytes (dotted arrow) of STs. E) AlCl_3_, VD and VCO-treated group showing an increase in expression of Ki-67 in the nuclei of spermatogonia (arrow) and spermatocytes. F) Statistical analysis of the immuno-expression % of Ki-67 in spermatogenic cells (dotted arrow) of STs. [Ki-67 immunostaining x 200].

As seen in Fig. 9, positive expression of Ki-67 proliferation marker in various spermatogenic cells (mainly spermatogonia and spermatocytes as a brown coloration) of control, VD and VCO groups showed strong staining, indicating active spermatogenesis with no staining was found in Leydig or Sertoli cells. In contrast, negative or weak ki-67 expression was detected in the nuclei of these cells in AlCl_3_ treated rats, which indicated depletion in the cell proliferation. However, co-administration of VD and VCO with AlCl_3_ restored Ki-67 immunostaining to a pattern similar to the control group. Additionally, a statistical analysis of the immuno-expression % of Ki-67 in spermatogenic cells showed that the AlCl_3_ treated group had a considerable increase in this % (P < 0.0001) in comparison to control groups, while the AlCl_3_ co-treated groups with VD and VCO had a substantial decline in this % (P < 0.01). Moreover, the AlCl_3_ group combined VD and VCO co-treatment brought the % back to control value (P < 0.001).

### 3.8 Morphometric results

**Fig. 10:**
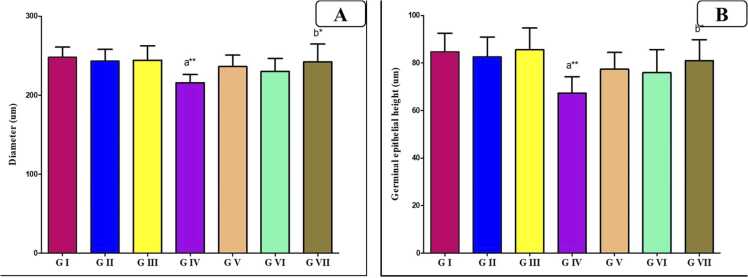
Bar charts showing the statistical comparison of the mean values ± SD of morphometric parameters between different studied groups (*n* = 8): (A) diameter of seminiferous tubules, (B) height of germinal epithelium of the seminiferous tubules. a: Significance versus GI, b: significance versus GIV. * : P < 0.05, * *: P < 0.01, * ** : P < 0.001. GI = control group, GII = VD treated group, GIII = VCO treated group, GIV = AlCl_3_ group, GV = AlCl_3_ and VD group, GVI = AlCl_3_ and VCO group, GVII = AlCl_3_, VD and VCO group.

Fig. 10 showed that AlCl_3_ treatment resulted in degeneration and necrosis, significantly reducing both the diameter of STc and height of the germinal epithelium by comparing to control group (P < 0.01 & P < 0.05). However, when either VD or VCO were co-administered individually with Al, these parameters were restored toward control values (P < 0.05), showing significance compared to Al-treated group. Furthermore, the administration of two agents together to Al-treated group led to furthermore improvement (P < 0.01), approaching the control values.

## 4 Discussion

This study aimed to explore the protective role of VD and VCO against AlCl_3_-induced testicular damage, given the well-established detrimental effects of Al on male reproductive health [36], [35] and the antioxidant properties of VD and VCO [19].

In this study, AlCl_3_ treated rats displayed a considerable reduction in body, testicular weights and gonadosomatic index; likely due to suppressed appetite, impaired nutrient absorption, and hormonal disturbances as reported by some earlier studies [26], [36].

In this study, the hormonal analyses revealed marked declines in testosterone, LH, and FSH levels in AlCl_3_-treated rats, in line with prior findings [8], [54]. These hormonal disruptions are multifactorial: AlCl3 blocks calcium channels, hindering gonadotropin release from the pituitary gland [3], and accumulates in Leydig cells, impairing testosterone synthesis [34]. Testosterone’s pivotal role in spermatogenesis underscores the reproductive implications of these findings [40].

Notably, VD and VCO supplementation, particularly in combination reversed these effects, restoring hormonal balance. VD has been shown to reduce oxidative stress and support germ cell proliferation [14], [28], while VCO’s bioactive compounds contribute to hormonal regulation and antioxidant defense [5], [16].

In current study, AlCl_3_ treatment caused a considerable rising in testicular Al concentration compared to both the control and protected groups. In accord, Berihu (2015) has mentioned that increased consumption of Al-containing products leads to elevated Al levels in consumers’ organs, potentially damaging various tissues, including the testes. This increased Al concentration caused male reproductive toxicity, possibly mediated through oxidative stress, interfering with spermatogenesis and steroidogenesis, and necrosis of spermatocytes/ spermatids. Furthermore, it may result in a significant decrease in fertility, as reported by Zhu et al. [58] and Martinez et al. (2017).

A central mechanism of Al toxicity involves oxidative stress. This study demonstrated elevated malondialdehyde (MDA) levels and suppressed antioxidant enzyme activities (SOD, CAT, GPx) in AlCl_3_-treated rats, consistent with earlier findings [34]; Güvenç et al., 2020). Excessive reactive oxygen species (ROS) production, mitochondrial dysfunction, and lipid peroxidation further disrupt spermatogenesis and Leydig cell steroidogenesis [32], [53]. On the other side, AlCl_3_ group co-treated with either VD or VCO resulted in an improvement in the oxidative stress with concomitant rise of antioxidant enzymes activity with the combined effect of both agents induced a more potent effect. In accordance, it was reported that VD has anti-oxidative effect potential [47]. VD triggers the formation of various molecules that are involved in the antioxidant protection, including GSH, GPx and SOD. Additionally, studies have reported that VD treatment significantly reduces oxidative stress, potentially decreasing apoptosis and promoting germ cell proliferation in aged testes [28], [50].

Also, AlCl_3_ administration significantly elevates testicular inflammatory markers, including NF-κB, TNF-α, IL-1β, and IL-6, corroborating previous findings that AlCl_3_ induces oxidative stress and inflammation in testicular tissues. The observed increase in these markers suggests that AlCl_3_ disrupts testicular homeostasis, potentially impairing spermatogenesis and steroidogenesis. However, VD supplementation in AlCl_3_-exposed rats resulted in a notable reduction of inflammatory markers. This aligns with prior studies indicating that VD exerts anti-inflammatory effects by modulating NF-κB signaling pathways. Additionally, VD has been shown to enhance antioxidant defenses, thereby mitigating oxidative stress-induced damage in testicular tissues [31], [1]. Similarly, VCO administration ameliorated AlCl_3_-induced testicular inflammation. The antioxidant properties of VCO, attributed to its high content of medium-chain fatty acids and phenolic compounds, have been reported to reduce oxidative stress and improve testicular histology in toxin-exposed models [20], [51].

In this study, administration of AlCl_3_ to rats caused marked disruption of testicular architecture, characterized by degeneration at various stages of the seminiferous tubules (STs), thinning of the germinal epithelium, and a notable reduction in the number of mature sperm within the lumina. Additionally, prominent interstitial edema and tissue enlargement were observed. These alterations are consistent with previous studies reporting structural damage following AlCl_3_ exposure, primarily due to excessive aluminum deposition within the testicular tissue. The pathological changes are likely attributable to oxidative stress, disruption of the blood-testis barrier, enhanced lipid peroxidation, and consequent damage to testicular membranes. Moreover, AlCl_3_ exposure induced degenerative changes in spermatogenic cells, including their detachment from the basement membrane of the STs, which was accompanied by vascular congestion and interstitial exudation. These findings corroborate earlier research describing similar histopathological alterations linked to prolonged aluminum accumulation in the testes [46]. Furthermore, the current investigation revealed significant dilation of the interstitial spaces, associated with the presence of excessive acidophilic vacuolated material. This observation aligns with findings by Akinola et al. [4], who reported that chronic AlCl_3_ administration led to pronounced widening of the interstitial spaces, attributed to necrosis, vascular congestion, and the accumulation of diffuse eosinophilic, edematous fluid.

Morphometric analysis revealed a significant reduction in both the diameter and epithelial height of the seminiferous tubules in the AlCl_3_-treated group compared to controls. However, co-treatment with VD or VCO markedly improved these structural parameters. These findings are consistent with previous studies demonstrating the toxic effects of Al on ST architecture in rats and mice [30], [11]. In support of these observations, VD has been reported to exert protective effects on STs, and similarly, VCO has been shown to preserve ST diameter and epithelial thickness in models of ethanol-induced testicular injury [44].

Conversely, co-treatment with either VD or VCO in AlCl_3_ administered rats led to a marked improvement in histopathological alterations and a regression of testicular fibrosis compared to the untreated AlCl₃ group. In terms of testicular health, VD has been shown to alleviate testicular damage by reducing seminiferous tubule degeneration and interstitial edema in models of imidacloprid-induced injury, as well as enhancing both function and structural integrity in diabetic rats [31]. In contrast, VD deficiency is associated with degeneration of the germinal epithelium and significant reductions in sperm counts, resulting in impaired testicular development and disrupted spermatogenesis [33], [56]. Similarly, VCO treatment demonstrated protective effects on the ST epithelium, evidenced by reduced degeneration and restoration of the normal epithelial architecture following AlCl₃ exposure. Supporting this, Ogedengbe et al. [38] reported that VCO administration at a dose of 10 ml/kg significantly ameliorated testicular injury.

The immunohistochemical analysis revealed that AlCl_3_ administration significantly enhanced cell death in the testes, as evidenced by increased immunostaining intensity for the pro-apoptotic protein caspase-3 within spermatogenic cells of the seminiferous tubules (STs), compared to controls. These findings are consistent with previous studies highlighting the cytotoxic effects of aluminum on rat testes, leading to elevated apoptosis among spermatogenic cells [32], 66. Apoptosis plays a fundamental role in maintaining spermatogenesis; however, excessive apoptotic activity within testicular tissues disrupts this balance and contributes to male infertility. Mechanistically, this process may be initiated by oxidative stress-induced DNA damage, resulting in mitochondrial membrane disruption, cytochrome-C release, and activation of caspase-mediated apoptotic pathways.

Similarly, Ki-67 immunostaining was employed in this study to assess proliferative activity within spermatogenic cells. A marked reduction in Ki-67 expression was observed in the seminiferous tubules (STs) of AlCl_3_-treated rats, indicating impaired cell proliferation and increased germ cell death. These findings are consistent with previous reports linking Ki-67 expression to the proliferative capacity of spermatogenic cells [57]. Conversely, co-treatment with VD or VCO attenuated apoptotic changes and enhanced spermatogenic cell proliferation, as evidenced by the modulation of caspase-3 and Ki-67 expression levels. Maintaining a balance between germ cell proliferation and apoptosis is essential for normal spermatogenesis [24], and in this context, VD has been demonstrated to exert anti-apoptotic effects by downregulating caspase-3 expression [28].

In conclusion, AlCl_3_ exposure significantly impaired testicular function, reducing body weight gain, testis weights, reproductive hormones, and antioxidant defenses, while increasing oxidative stress and testicular damage. Co-treatment with VD, VCO, or their combination effectively mitigated these effects, with the combined therapy offering the strongest protection. This was evidenced by restored hormone levels, improved antioxidant status, reduced tissue damage, and normalized expression of apoptotic (caspase-3) and proliferative (Ki-67) markers. Collectively, these findings suggest that VD and VCO synergistically counteract AlCl_3_-induced testicular toxicity, offering anti-inflammatory, antioxidant, anti-apoptotic, and proliferative protective mechanisms.

## Declaration of Competing Interest

The authors declare that they have no known competing financial interests or personal relationships that could have appeared to influence the work reported in this paper.

## Data Availability

Data will be made available on request.
